# Supplementary material for: Impact of Active Video Games on Body Mass Index in Children and Adolescents: Systematic Review and Meta-Analysis Evaluating the Quality of Primary Studies
Source: Int J Environ Res Public Health. 2019 Jul 8;16(13):2424. doi: 10.3390/ijerph16132424 (PMC6650803; doi:10.3390/ijerph16132424)
Supplement: Supplementary file 1 [file ijerph-16-02424-s001.pdf]

Supplementary Material

S1 Figure. Pre-post intra-group difference in BMI in the group intervened with active video games. Subgroup analysis based on the quantitative assessment of the quality of the primary studies.

Model	Group by Methodological quality second approach	Study name		Outcome	Std diff in means	Standard error	Variance	Lower limit	Upper limit	Z-Value	p-Value
	<8	Adamo 2010	10 weeks	BMI (Kg/m2)	0.000	0.392	0.154	-0.769	0.769	0.000	1.000
	<8	Azevedo 2014	50 weeks	BMI (Kg/m2)	0.257	0.118	0.014	0.025	0.489	2.171	0.030
	<8	Bethea 2012	12 weeks	BMI (Kg/m2)	0.026	0.275	0.076	-0.514	0.565	0.093	0.926
	<8	Bethea 2012	30 weeks	BMI (Kg/m2)	-0.054	0.281	0.079	-0.605	0.498	-0.191	0.849
	<8	Calcaterra 2013	12 weeks	BMI (Kg/m2)	-0.225	0.302	0.091	-0.817	0.368	-0.743	0.458
	<8	Christison 2012	10 weeks	BMI (Kg/m2)	-0.076	0.224	0.050	-0.514	0.363	-0.339	0.735
	<8	Fernandez-Rosado	13 weeks	BMI (Kg/m2)	-0.673	0.375	0.141	-1.409	0.062	-1.794	0.073
	<8	Graves 2010	6 weeks	BMI (Kg/m2)	-0.022	0.302	0.091	-0.613	0.569	-0.074	0.941
	<8	Graves 2010	12 weeks	BMI (Kg/m2)	0.000	0.302	0.091	-0.591	0.591	0.000	1.000
	<8	Lau 2016	12 weeks	BMI (Kg/m2)	0.146	0.224	0.050	-0.293	0.585	0.651	0.515
	<8	Murphy 2009	12 weeks	BMI (Kg/m2)	-0.021	0.295	0.087	-0.599	0.557	-0.071	0.943
	<8	Owens 2011	13 weeks	BMI (Kg/m2)	0.045	0.408	0.167	-0.755	0.846	0.111	0.911
Fixed	<8				0.059	0.071	0.005	-0.079	0.198	0.841	0.400
Random	<8				0.059	0.071	0.005	-0.079	0.198	0.841	0.400
	=>8	Christison 2016	25 weeks	BMI (Kg/m2)	0.015	0.213	0.046	-0.403	0.434	0.072	0.942
	=>8	Maddison 2011	12 weeks	BMI (Kg/m2)	-0.173	0.128	0.016	-0.423	0.078	-1.353	0.176
	=>8	Maddison 2011	24 weeks	BMI (Kg/m2)	-0.207	0.128	0.016	-0.458	0.043	-1.622	0.105
	=>8	Staiano2017	12 weeks	BMI z-score	-0.004	0.309	0.095	-0.610	0.602	-0.013	0.990
	=>8	Trost 2013	8 weeks	BMI z-score	-1.625	0.293	0.086	-2.199	-1.051	-5.547	0.000
	=>8	Trost 2013	16 weeks	BMI z-score	-3.125	0.379	0.143	-3.867	-2.383	-8.256	0.000
	=>8	Wagener 2012	10 weeks	BMI z-score	-0.108	0.316	0.100	-0.728	0.512	-0.341	0.733
Fixed	=>8				-0.353	0.074	0.005	-0.498	-0.208	-4.784	0.000
Random	=>8				-0.696	0.295	0.087	-1.275	-0.117	-2.358	0.018
Fixed	Overall				-0.138	0.051	0.003	-0.237	-0.038	-2.696	0.007
Random	Overall				0.019	0.069	0.005	-0.116	0.153	0.270	0.787

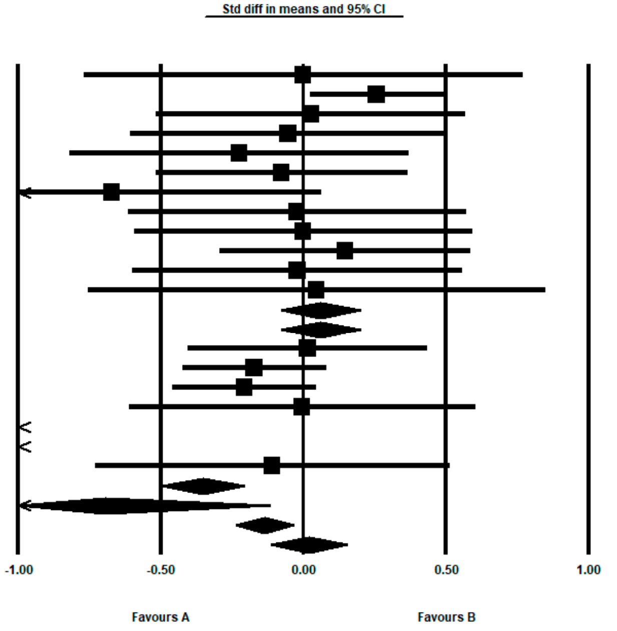

S2 Figure. Pre-post intra-group difference in BMI in the group intervened with active video games. Analysis of subgroups according to their publication date (last 5 years versus ≤2010).

Model	Group by Publication date.	Study name		Outcome	Std diff in means	Standard error	Variance	Lower limit	Upper limit	Z-Value	p-Value
	<=2010	Adamo 2010	10 weeks	BMI (Kg/m2)	0.000	0.392	0.154	-0.769	0.769	0.000	1.000
	<=2010	Graves 2010	6 weeks	BMI (Kg/m2)	-0.022	0.302	0.091	-0.613	0.569	-0.074	0.941
	<=2010	Graves 2010	12 weeks	BMI (Kg/m2)	0.000	0.302	0.091	-0.591	0.591	0.000	1.000
	<=2010	Murphy 2009	12 weeks	BMI (Kg/m2)	-0.021	0.295	0.087	-0.599	0.557	-0.071	0.943
Fixed	<=2010				-0.012	0.158	0.025	-0.322	0.298	-0.077	0.939
Random	<=2010				-0.012	0.158	0.025	-0.322	0.298	-0.077	0.939
	>2010	Azevedo 2014	50 weeks	BMI (Kg/m2)	0.257	0.118	0.014	0.025	0.489	2.171	0.030
	>2010	Bethea 2012	12 weeks	BMI (Kg/m2)	0.026	0.275	0.076	-0.514	0.565	0.093	0.926
	>2010	Bethea 2012	30 weeks	BMI (Kg/m2)	-0.054	0.281	0.079	-0.605	0.498	-0.191	0.849
	>2010	Calcaterra 2013	12 weeks	BMI (Kg/m2)	-0.225	0.302	0.091	-0.817	0.368	-0.743	0.458
	>2010	Christison 2012	10 weeks	BMI (Kg/m2)	-0.076	0.224	0.050	-0.514	0.363	-0.339	0.735
	>2010	Christison 2016	25 weeks	BMI (Kg/m2)	0.015	0.213	0.046	-0.403	0.434	0.072	0.942
	>2010	Fernandez-Rosado	13 weeks	BMI (Kg/m2)	-0.673	0.375	0.141	-1.409	0.062	-1.794	0.073
	>2010	Lau 2016	12 weeks	BMI (Kg/m2)	0.146	0.224	0.050	-0.293	0.585	0.651	0.515
	>2010	Maddison 2011	12 weeks	BMI (Kg/m2)	-0.173	0.128	0.016	-0.423	0.078	-1.353	0.176
	>2010	Maddison 2011	24 weeks	BMI (Kg/m2)	-0.207	0.128	0.016	-0.458	0.043	-1.622	0.105
	>2010	Owens 2011	13 weeks	BMI (Kg/m2)	0.045	0.408	0.167	-0.755	0.846	0.111	0.911
	>2010	Staiano2017	12 weeks	BMI z-score	-0.004	0.309	0.095	-0.610	0.602	-0.013	0.990
	>2010	Trost 2013	8 weeks	BMI z-score	-1.625	0.293	0.086	-2.199	-1.051	-5.547	0.000
	>2010	Trost 2013	16 weeks	BMI z-score	-3.125	0.379	0.143	-3.867	-2.383	-8.256	0.000
	>2010	Wagener 2012	10 weeks	BMI z-score	-0.108	0.316	0.100	-0.728	0.512	-0.341	0.733
Fixed	>2010				-0.152	0.054	0.003	-0.258	-0.046	-2.822	0.005
Random	>2010				-0.342	0.158	0.025	-0.651	-0.033	-2.167	0.030
Fixed	Overall				-0.138	0.051	0.003	-0.237	-0.038	-2.696	0.007
Random	Overall				-0.177	0.112	0.012	-0.396	0.041	-1.589	0.112

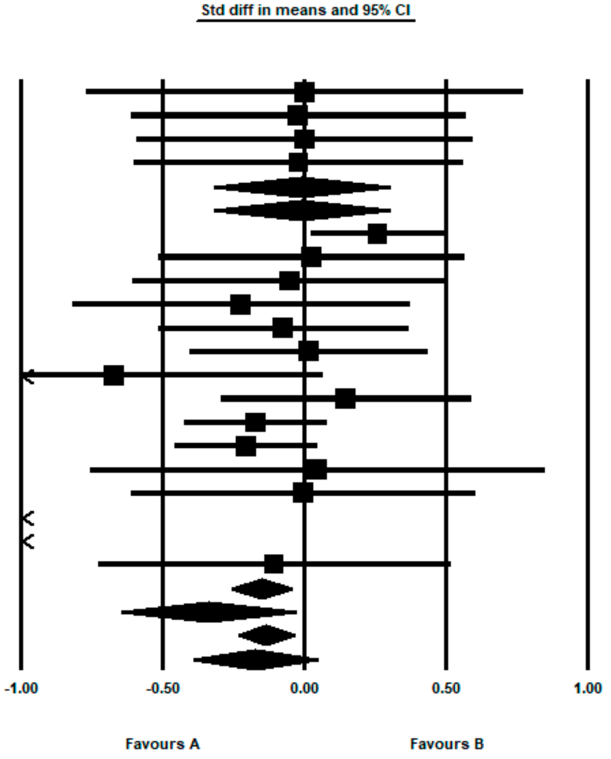

S3 Figure. Pre-post intra-group difference in BMI in the group intervened with active video games. Analysis of subgroups according to their place of study (Europe, USA, Australia & New Zealand).

Model	Group by Location.	Study name		Outcome	Std diff in means	Standard error	Variance	Lower limit	Upper limit	Z-Value	p-Value
	Asia	Lau 2016	12 weeks	BMI (Kg/m2)	0.146	0.224	0.050	-0.293	0.585	0.651	0.515
Fixed	Asia				0.146	0.224	0.050	-0.293	0.585	0.651	0.515
Random	Asia				0.146	0.224	0.050	-0.293	0.585	0.651	0.515
	Australia&Nueva Zelanda	Maddison 2011	12 weeks	BMI (Kg/m2)	-0.173	0.128	0.016	-0.423	0.078	-1.353	0.176
	Australia&Nueva Zelanda	Maddison 2011	24 weeks	BMI (Kg/m2)	-0.207	0.128	0.016	-0.458	0.043	-1.622	0.105
Fixed	Australia&Nueva Zelanda				-0.190	0.090	0.008	-0.367	-0.013	-2.103	0.035
Random	Australia&Nueva Zelanda				-0.190	0.090	0.008	-0.367	-0.013	-2.103	0.035
	EEUU&Canada	Adamo 2010	10 weeks	BMI (Kg/m2)	0.000	0.392	0.154	-0.769	0.769	0.000	1.000
	EEUU&Canada	Bethea 2012	12 weeks	BMI (Kg/m2)	0.026	0.275	0.076	-0.514	0.565	0.093	0.926
	EEUU&Canada	Bethea 2012	30 weeks	BMI (Kg/m2)	-0.054	0.281	0.079	-0.605	0.498	-0.191	0.849
	EEUU&Canada	Christison 2012	10 weeks	BMI (Kg/m2)	-0.076	0.224	0.050	-0.514	0.363	-0.339	0.735
	EEUU&Canada	Christison 2016	25 weeks	BMI (Kg/m2)	0.015	0.213	0.046	-0.403	0.434	0.072	0.942
	EEUU&Canada	Murphy 2009	12 weeks	BMI (Kg/m2)	-0.021	0.295	0.087	-0.599	0.557	-0.071	0.943
	EEUU&Canada	Owens 2011	13 weeks	BMI (Kg/m2)	0.045	0.408	0.167	-0.755	0.846	0.111	0.911
	EEUU&Canada	Staiano2017	12 weeks	BMI z-score	-0.004	0.309	0.095	-0.610	0.602	-0.013	0.990
	EEUU&Canada	Trost 2013	8 weeks	BMI z-score	-1.625	0.293	0.086	-2.199	-1.051	-5.547	0.000
	EEUU&Canada	Trost 2013	16 weeks	BMI z-score	-3.125	0.379	0.143	-3.867	-2.383	-8.256	0.000
	EEUU&Canada	Wagener 2012	10 weeks	BMI z-score	-0.108	0.316	0.100	-0.728	0.512	-0.341	0.733
Fixed	EEUU&Canada				-0.331	0.087	0.008	-0.503	-0.160	-3.790	0.000
Random	EEUU&Canada				-0.434	0.259	0.067	-0.941	0.073	-1.676	0.094
	Europa	Azevedo 2014	50 weeks	BMI (Kg/m2)	0.257	0.118	0.014	0.025	0.489	2.171	0.030
	Europa	Calcaterra 2013	12 weeks	BMI (Kg/m2)	-0.225	0.302	0.091	-0.817	0.368	-0.743	0.458
	Europa	Fernandez-Rosado	13 weeks	BMI (Kg/m2)	-0.673	0.375	0.141	-1.409	0.062	-1.794	0.073
	Europa	Graves 2010	6 weeks	BMI (Kg/m2)	-0.022	0.302	0.091	-0.613	0.569	-0.074	0.941
	Europa	Graves 2010	12 weeks	BMI (Kg/m2)	0.000	0.302	0.091	-0.591	0.591	0.000	1.000
Fixed	Europa				0.097	0.095	0.009	-0.088	0.283	1.028	0.304
Random	Europa				-0.035	0.158	0.025	-0.345	0.275	-0.223	0.824
Fixed	Overall				-0.138	0.051	0.003	-0.237	-0.038	-2.696	0.007
Random	Overall				-0.143	0.071	0.005	-0.283	-0.004	-2.011	0.044

Std diff in means and 95% CI

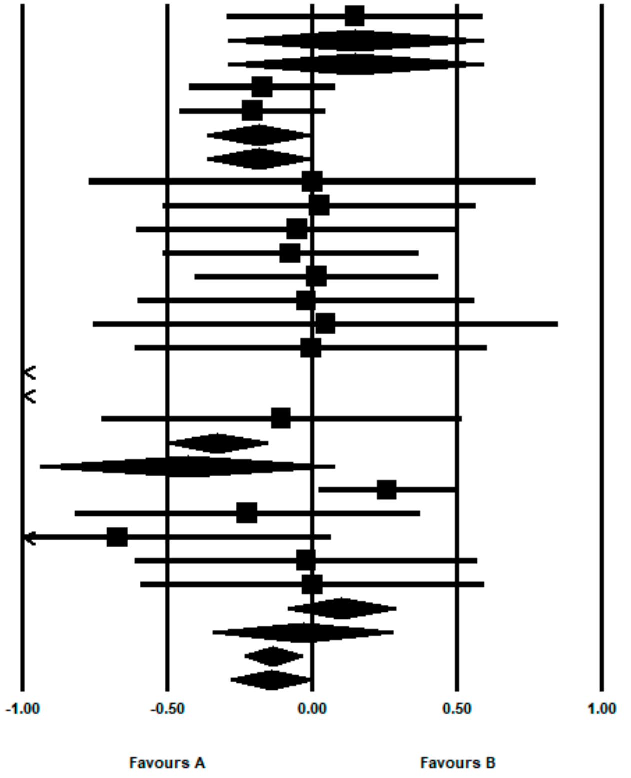

S4 Figure. Pre-post intra-group difference in BMI in the group intervened with active video games. Subgroup analysis based on the design of the controlled studies (randomized vs. non-randomized).

Model	Group by Randomized	Study name		Outcome	Std diff in means	Standard error	Variance	Lower limit	Upper limit	Z-Value	p-Value
	No	Azevedo 2014	50 weeks	BMI (Kg/m2)	0.257	0.118	0.014	0.025	0.489	2.171	0.030
Fixed	No				0.257	0.118	0.014	0.025	0.489	2.171	0.030
Random	No				0.257	0.118	0.014	0.025	0.489	2.171	0.030
	Yes	Adamo 2010	10 weeks	BMI (Kg/m2)	0.000	0.392	0.154	-0.769	0.769	0.000	1.000
	Yes	Christison 2016	25 weeks	BMI (Kg/m2)	0.015	0.213	0.046	-0.403	0.434	0.072	0.942
	Yes	Graves 2010	6 weeks	BMI (Kg/m2)	-0.022	0.302	0.091	-0.613	0.569	-0.074	0.941
	Yes	Graves 2010	12 weeks	BMI (Kg/m2)	0.000	0.302	0.091	-0.591	0.591	0.000	1.000
	Yes	Lau 2016	12 weeks	BMI (Kg/m2)	0.146	0.224	0.050	-0.293	0.585	0.651	0.515
	Yes	Maddison 2011	12 weeks	BMI (Kg/m2)	-0.173	0.128	0.016	-0.423	0.078	-1.353	0.176
	Yes	Maddison 2011	24 weeks	BMI (Kg/m2)	-0.207	0.128	0.016	-0.458	0.043	-1.622	0.105
	Yes	Murphy 2009	12 weeks	BMI (Kg/m2)	-0.021	0.295	0.087	-0.599	0.557	-0.071	0.943
	Yes	Staiano2017	12 weeks	BMI z-score	-0.004	0.309	0.095	-0.610	0.602	-0.013	0.990
	Yes	Trost 2013	8 weeks	BMI z-score	-1.625	0.293	0.086	-2.199	-1.051	-5.547	0.000
	Yes	Trost 2013	16 weeks	BMI z-score	-3.125	0.379	0.143	-3.867	-2.383	-8.256	0.000
	Yes	Wagener 2012	10 weeks	BMI z-score	-0.108	0.316	0.100	-0.728	0.512	-0.341	0.733
Fixed	Yes				-0.256	0.064	0.004	-0.382	-0.131	-3.998	0.000
Random	Yes				-0.395	0.194	0.038	-0.775	-0.015	-2.036	0.042
Fixed	Overall				-0.140	0.056	0.003	-0.250	-0.030	-2.484	0.013
Random	Overall				0.080	0.101	0.010	-0.118	0.278	0.791	0.429

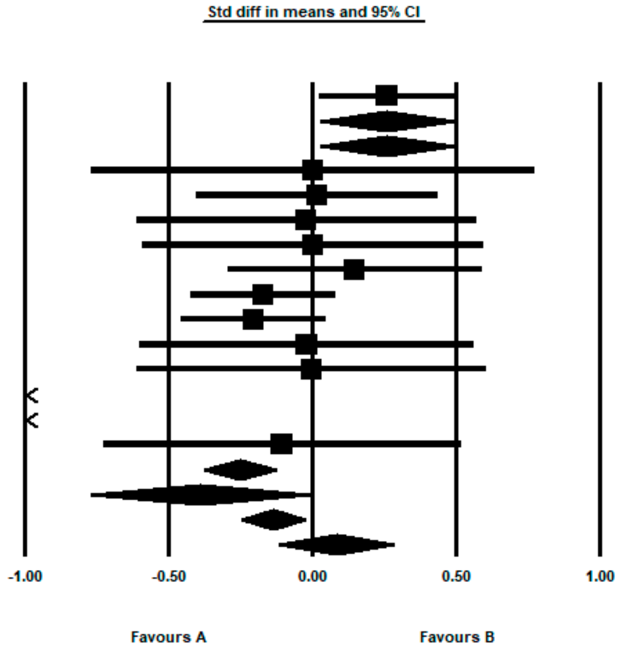

S5 Figure. Pre-post intra-group difference in BMI in the group intervened with active video games. Analysis of subgroups according to the weeks of follow-up: '(<12 weeks) (≥12- <24 weeks) (≥24 weeks)'.

Model	Group by Weeks.	Study name		Outcome	Std diff in means	Standard error	Variance	Lower limit	Upper limit	Z-Value	p-Value
	<12	Adamo 2010	10 weeks	BMI (Kg/m2)	0.000	0.392	0.154	-0.769	0.769	0.000	1.000
	<12	Christison 2012	10 weeks	BMI (Kg/m2)	-0.076	0.224	0.050	-0.514	0.363	-0.339	0.735
	<12	Graves 2010	6 weeks	BMI (Kg/m2)	-0.022	0.302	0.091	-0.613	0.569	-0.074	0.941
	<12	Trost 2013	8 weeks	BMI z-score	-1.625	0.293	0.086	-2.199	-1.051	-5.547	0.000
	<12	Wagener 2012	10 weeks	BMI z-score	-0.108	0.316	0.100	-0.728	0.512	-0.341	0.733
Fixed	<12				-0.368	0.130	0.017	-0.623	-0.113	-2.832	0.005
Random	<12				-0.373	0.319	0.102	-0.999	0.253	-1.167	0.243
	>=12 <24	Bethea 2012	12 weeks	BMI (Kg/m2)	0.026	0.275	0.076	-0.514	0.565	0.093	0.926
	>=12 <24	Calcaterra 2013	12 weeks	BMI (Kg/m2)	-0.225	0.302	0.091	-0.817	0.368	-0.743	0.458
	>=12 <24	Fernandez-Rosado	13 weeks	BMI (Kg/m2)	-0.673	0.375	0.141	-1.409	0.062	-1.794	0.073
	>=12 <24	Graves 2010	12 weeks	BMI (Kg/m2)	0.000	0.302	0.091	-0.591	0.591	0.000	1.000
	>=12 <24	Lau 2016	12 weeks	BMI (Kg/m2)	0.146	0.224	0.050	-0.293	0.585	0.651	0.515
	>=12 <24	Maddison 2011	12 weeks	BMI (Kg/m2)	-0.173	0.128	0.016	-0.423	0.078	-1.353	0.176
	>=12 <24	Murphy 2009	12 weeks	BMI (Kg/m2)	-0.021	0.295	0.087	-0.599	0.557	-0.071	0.943
	>=12 <24	Owens 2011	13 weeks	BMI (Kg/m2)	0.045	0.408	0.167	-0.755	0.846	0.111	0.911
	>=12 <24	Staiano2017	12 weeks	BMI z-score	-0.004	0.309	0.095	-0.610	0.602	-0.013	0.990
	>=12 <24	Trost 2013	16 weeks	BMI z-score	-3.125	0.379	0.143	-3.867	-2.383	-8.256	0.000
Fixed	>=12 <24				-0.230	0.079	0.006	-0.386	-0.074	-2.892	0.004
Random	>=12 <24				-0.370	0.232	0.054	-0.825	0.085	-1.593	0.111
	>=24	Azevedo 2014	50 weeks	BMI (Kg/m2)	0.257	0.118	0.014	0.025	0.489	2.171	0.030
	>=24	Bethea 2012	30 weeks	BMI (Kg/m2)	-0.054	0.281	0.079	-0.605	0.498	-0.191	0.849
	>=24	Christison 2016	25 weeks	BMI (Kg/m2)	0.015	0.213	0.046	-0.403	0.434	0.072	0.942
	>=24	Maddison 2011	24 weeks	BMI (Kg/m2)	-0.207	0.128	0.016	-0.458	0.043	-1.622	0.105
Fixed	>=24				0.032	0.077	0.006	-0.120	0.183	0.410	0.682
Random	>=24				0.015	0.131	0.017	-0.243	0.272	0.112	0.911
Fixed	Overall				-0.138	0.051	0.003	-0.237	-0.038	-2.696	0.007
Random	Overall				-0.112	0.108	0.012	-0.323	0.099	-1.040	0.298

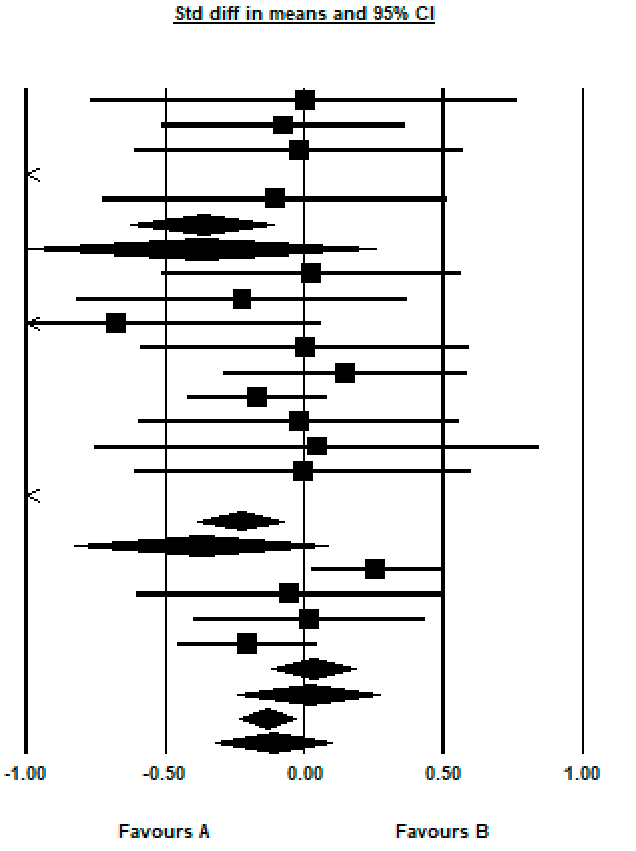

S6 Figure. Pre-post intra-group difference in BMI in the group intervened with active video games. Analysis of subgroups according to the minimum age of children or adolescents (<12 years vs ≥12).

Model	Group by Age	Study name		Outcome	Std diff in means	Standard error	Variance	Lower limit	Upper limit	Z-Value	p-Value
					Statistics for each study						
	<12	Azevedo 2014	50 weeks	BMI (Kg/m2)	0.257	0.118	0.014	0.025	0.489	2.171	0.030
	<12	Bethea 2012	12 weeks	BMI (Kg/m2)	0.026	0.275	0.076	-0.514	0.565	0.093	0.926
	<12	Bethea 2012	30 weeks	BMI (Kg/m2)	-0.054	0.281	0.079	-0.605	0.498	-0.191	0.849
	<12	Calcaterra 2013	12 weeks	BMI (Kg/m2)	-0.225	0.302	0.091	-0.817	0.368	-0.743	0.458
	<12	Christison 2012	10 weeks	BMI (Kg/m2)	-0.076	0.224	0.050	-0.514	0.363	-0.339	0.735
	<12	Christison 2016	25 weeks	BMI (Kg/m2)	0.015	0.213	0.046	-0.403	0.434	0.072	0.942
	<12	Fernandez-Rosado	13 weeks	BMI (Kg/m2)	-0.673	0.375	0.141	-1.409	0.062	-1.794	0.073
	<12	Graves 2010	6 weeks	BMI (Kg/m2)	-0.022	0.302	0.091	-0.613	0.569	-0.074	0.941
	<12	Graves 2010	12 weeks	BMI (Kg/m2)	0.000	0.302	0.091	-0.591	0.591	0.000	1.000
	<12	Lau 2016	12 weeks	BMI (Kg/m2)	0.146	0.224	0.050	-0.293	0.585	0.651	0.515
	<12	Maddison 2011	12 weeks	BMI (Kg/m2)	-0.173	0.128	0.016	-0.423	0.078	-1.353	0.176
	<12	Maddison 2011	24 weeks	BMI (Kg/m2)	-0.207	0.128	0.016	-0.458	0.043	-1.622	0.105
	<12	Murphy 2009	12 weeks	BMI (Kg/m2)	-0.021	0.295	0.087	-0.599	0.557	-0.071	0.943
	<12	Owens 2011	13 weeks	BMI (Kg/m2)	0.045	0.408	0.167	-0.755	0.846	0.111	0.911
	<12	Trost 2013	8 weeks	BMI z-score	-1.625	0.293	0.086	-2.199	-1.051	-5.547	0.000
	<12	Trost 2013	16 weeks	BMI z-score	-3.125	0.379	0.143	-3.867	-2.383	-8.256	0.000
Fixed	<12				-0.145	0.053	0.003	-0.248	-0.041	-2.737	0.006
Random	<12				-0.315	0.149	0.022	-0.608	-0.022	-2.107	0.035
	>=12	Adamo 2010	10 weeks	BMI (Kg/m2)	0.000	0.392	0.154	-0.769	0.769	0.000	1.000
	>=12	Staiano2017	12 weeks	BMI z-score	-0.004	0.309	0.095	-0.610	0.602	-0.013	0.990
	>=12	Wagener 2012	10 weeks	BMI z-score	-0.108	0.316	0.100	-0.728	0.512	-0.341	0.733
Fixed	>=12				-0.042	0.193	0.037	-0.419	0.336	-0.216	0.829
Random	>=12				-0.042	0.193	0.037	-0.419	0.336	-0.216	0.829
Fixed	Overall				-0.138	0.051	0.003	-0.237	-0.038	-2.696	0.007
Random	Overall				-0.212	0.118	0.014	-0.444	0.019	-1.797	0.072

Std diff in means and 95% CI

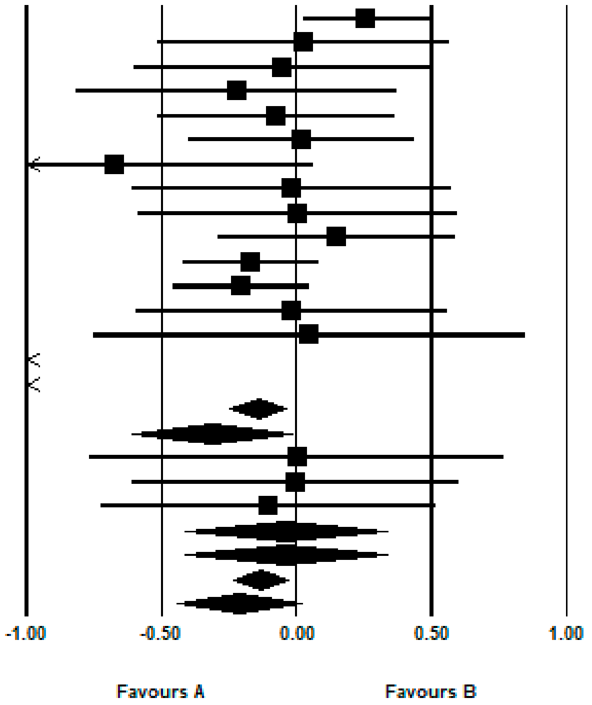

S7 Figure. Pre-post intra-group difference in BMI in the group intervened with active video games. Analysis of subgroups according to gender (male versus female versus ´both sexes or sex not specified´).

Model	Group by Gender	Study name		Outcome	Std diff in means	Standard error	Variance	Lower limit	Upper limit	Z-Value	p-Value
	Both	Adamo 2010	10 weeks	BMI (Kg/m2)	0.000	0.392	0.154	-0.769	0.769	0.000	1.000
	Both	Azevedo 2014	50 weeks	BMI (Kg/m2)	0.257	0.118	0.014	0.025	0.489	2.171	0.030
	Both	Calcaterra 2013	12 weeks	BMI (Kg/m2)	-0.225	0.302	0.091	-0.817	0.368	-0.743	0.458
	Both	Christison 2012	10 weeks	BMI (Kg/m2)	-0.076	0.224	0.050	-0.514	0.363	-0.339	0.735
	Both	Christison 2016	25 weeks	BMI (Kg/m2)	0.015	0.213	0.046	-0.403	0.434	0.072	0.942
	Both	Fernandez-Rosado	13 weeks	BMI (Kg/m2)	-0.673	0.375	0.141	-1.409	0.062	-1.794	0.073
	Both	Graves 2010	6 weeks	BMI (Kg/m2)	-0.022	0.302	0.091	-0.613	0.569	-0.074	0.941
	Both	Graves 2010	12 weeks	BMI (Kg/m2)	0.000	0.302	0.091	-0.591	0.591	0.000	1.000
	Both	Lau 2016	12 weeks	BMI (Kg/m2)	0.146	0.224	0.050	-0.293	0.585	0.651	0.515
	Both	Maddison 2011	12 weeks	BMI (Kg/m2)	-0.173	0.128	0.016	-0.423	0.078	-1.353	0.176
	Both	Maddison 2011	24 weeks	BMI (Kg/m2)	-0.207	0.128	0.016	-0.458	0.043	-1.622	0.105
	Both	Murphy 2009	12 weeks	BMI (Kg/m2)	-0.021	0.295	0.087	-0.599	0.557	-0.071	0.943
	Both	Owens 2011	13 weeks	BMI (Kg/m2)	0.045	0.408	0.167	-0.755	0.846	0.111	0.911
	Both	Trost 2013	8 weeks	BMI z-score	-1.625	0.293	0.086	-2.199	-1.051	-5.547	0.000
	Both	Trost 2013	16 weeks	BMI z-score	-3.125	0.379	0.143	-3.867	-2.383	-8.256	0.000
	Both	Wagener 2012	10 weeks	BMI z-score	-0.108	0.316	0.100	-0.728	0.512	-0.341	0.733
Fixed	Both				-0.151	0.054	0.003	-0.256	-0.046	-2.813	0.005
Random	Both				-0.325	0.152	0.023	-0.623	-0.027	-2.134	0.033
	Female	Staiano2017	12 weeks	BMI z-score	-0.004	0.309	0.095	-0.610	0.602	-0.013	0.990
Fixed	Female				-0.004	0.309	0.095	-0.610	0.602	-0.013	0.990
Random	Female				-0.004	0.309	0.095	-0.610	0.602	-0.013	0.990
	Male	Bethea 2012	12 weeks	BMI (Kg/m2)	0.026	0.275	0.076	-0.514	0.565	0.093	0.926
	Male	Bethea 2012	30 weeks	BMI (Kg/m2)	-0.054	0.281	0.079	-0.605	0.498	-0.191	0.849
Fixed	Male				-0.013	0.197	0.039	-0.399	0.373	-0.067	0.947
Random	Male				-0.013	0.197	0.039	-0.399	0.373	-0.067	0.947
Fixed	Overall				-0.138	0.051	0.003	-0.237	-0.038	-2.696	0.007
Random	Overall				-0.181	0.112	0.013	-0.401	0.039	-1.615	0.106

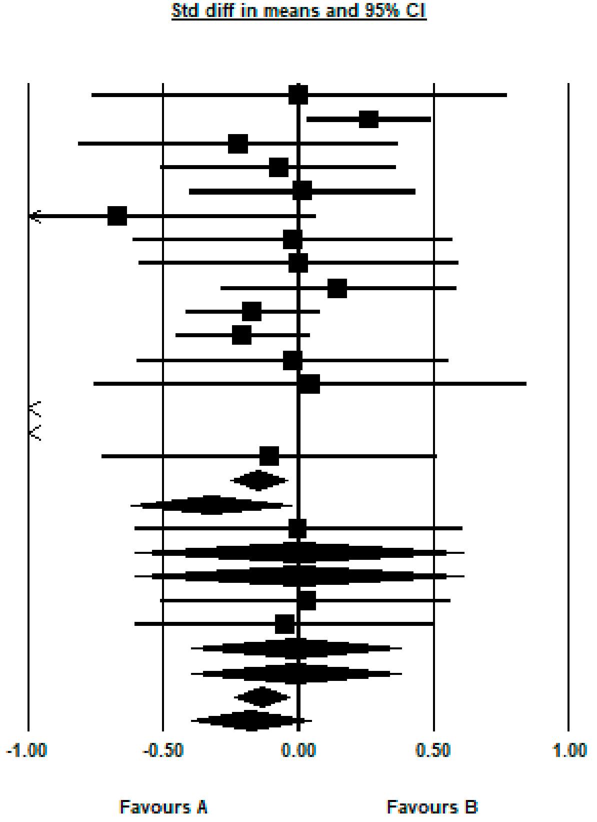

Author Contributions

CHJ and MS were responsible for the study conception and design, managed data collection and performed analysis. CHJ, RS, MPZ, PPB, and MS were responsible for manuscript preparation. All authors interpreted analyses and made critical revisions to the paper for important intellectual content. All authors read and approved the final version of manuscript.

Conflict of interest

All authors declare that they have no conflict of interest.

Funding

The authors received no specific funding for this work.

Data Availability

Datasets used and/or analysed during the current study available from the corresponding author on reasonable request.
